# Did online publishers “get it right”? Using a naturalistic search strategy to review cognitive health promotion content on internet webpages

**DOI:** 10.1186/s12877-017-0515-3

**Published:** 2017-06-15

**Authors:** P.V. Hunter, M. Delbaere, M. E. O’Connell, A. Cammer, J. X. Seaton, T. Friedrich, F. Fick

**Affiliations:** 1 St. Thomas More College, University of Saskatchewan, 1437 College Drive, Saskatoon, SK S7M 0W6 Canada; 2 Edwards School of Business, University of Saskatchewan, 25 Campus Drive, Saskatoon, SK S7N 5A7 Canada; 3 Psychology, University of Saskatchewan, 9 Campus Drive, Saskatoon, SK S7N 5A5 Canada; 4 College of Pharmacy and Nutrition, University of Saskatchewan, 110 Science Place, Saskatoon, SK S7N 5C9 Canada; 5 Interdisciplinary Studies, University of Saskatchewan, 176 Thorvaldson Building, 110 Science Place, Saskatoon, SK S7N 5C9 Canada

## Abstract

**Background:**

One of the most common uses of the Internet is to search for health-related information. Although scientific evidence pertaining to cognitive health promotion has expanded rapidly in recent years, it is unclear how much of this information has been made available to Internet users. Thus, the purpose of our study was to assess the reliability and quality of information about cognitive health promotion encountered by typical Internet users.

**Methods:**

To generate a list of relevant search terms employed by Internet users, we entered seed search terms in Google Trends and recorded any terms consistently used in the prior 2 years. To further approximate the behaviour of typical Internet users, we entered each term in Google and sampled the first two relevant results. This search, completed in October 2014, resulted in a sample of 86 webpages, 48 of which had content related to cognitive health promotion. An interdisciplinary team rated the information reliability and quality of these webpages using a standardized measure.

**Results:**

We found that information reliability and quality were moderate, on average. Just one retrieved page mentioned best practice, national recommendations, or consensus guidelines by name. Commercial content (i.e., product promotion, advertising content, or non-commercial) was associated with differences in reliability and quality, with product promoter webpages having the lowest mean reliability and quality ratings.

**Conclusions:**

As efforts to communicate the association between lifestyle and cognitive health continue to expand, we offer these results as a baseline assessment of the reliability and quality of cognitive health promotion on the Internet.

**Electronic supplementary material:**

The online version of this article (doi:10.1186/s12877-017-0515-3) contains supplementary material, which is available to authorized users.

## Background

Evidence is converging to suggest that individual behavioural choices may influence age-related changes in cognition. Epidemiological research suggests that among the most promising behavioural choices to support cognitive health include: managing cardiovascular risk factors; engaging in educational or other mentally stimulating activities; being physically active; developing social supports; using alcohol in moderation; avoiding use of tobacco; and eating a nourishing diet such as a Mediterranean diet [1, 2]. Since interactions between genetic factors and behavioural choices may convey different degrees of advantage to different individuals, [3, 4] fostering attention to multiple risk management and health promotion strategies is increasingly advocated [5–8]. Although there remains some hesitation about promoting such strategies on the basis of observational studies rather than randomized controlled trials, [9, 10] it is nevertheless estimated that reducing lifestyle-related risk factors by 10 to 25% in the human population has potential to prevent approximately 1 to 3 million cases of dementia worldwide [11].

Despite a more significant media focus on cognitive health promotion in recent years, surveys of the North American public suggest that misperceptions about the role of lifestyle in cognitive health continue. For instance, nearly half the participants in a large American survey believed that there is “nothing a person can do to help maintain a healthy memory and reduce the chance of Alzheimer's disease” [12]. Americans are also more likely to believe that doing crossword puzzles reduces risk of Alzheimer’s disease than to believe that engagement in regular physically activity reduces risk [13]. As the increasing prevalence of dementia continues to place pressure on health care and social systems, [14] gaps in public knowledge about strategies to promote cognitive health are concerning.

Social marketing campaigns have successfully promoted healthy behavioural choices to the general public for decades. For instance, the VERB™ campaign saw a 34% increase in the free-time physical activity of American pre-teens, [15] and the messages promoted in Canada’s long-lived ParticipACTION campaign were recognized by 85% of Canadians in 2002 [16]. Given considerable potential for well-constructed social marketing campaigns to influence lifestyle, North American governments and special interest groups are aligning themselves with a global movement [17, 18] to disseminate available evidence about promoting cognitive health and managing risk factors for cognitive decline to the wider public. For example, the USA Centers for Disease Control and Prevention are collaborating with the American Alzheimer’s Association and other organizations on a *Healthy Brain Initiative* to improve population cognitive health and support those with cognitive impairment [19]. Similarly, Alzheimer Society of Canada [14] has recommended government sponsorship of public health initiatives to promote lifestyle choices (e.g., engagement in physical activity and exercise, healthy diet) to reduce incident cases of Alzheimer’s disease.

Internet-based communication may be a useful component of public health campaigns and social marketing initiatives to improve brain health, since the Internet is widely used by North Americans to search for information related to health. For example, approximately 50% of Americans and nearly two thirds of Canadians use the Internet to search for this purpose, and this is one of the top uses for the Internet in Canada [20, 21]. There is also evidence that the public actively uses the Internet for health research across the adult lifespan [20, 21]. Despite these facts, reviews of the quality of Internet-based information about cognitive health are largely absent. One study reviewed the type of information provided by health care systems, public health departments, and senior centres in the United States to determine how much and what kind of information was provided about lifestyle-related strategies to promote cognitive health [22]. Of 70 health websites identified for review, only 40% provided information about strategies to promote cognitive health. Content was variable, and only half of these websites included any information about physical activity – arguably the best-evidenced strategy [22].

With a focus on health care systems, public health departments, and senior centres, this review, completed by Laditka and colleagues in 2012, [22] illustrated the range of information available across diverse regions or communities in the USA. Laditka’s approach yielded an excellent foundation upon which to develop public health strategies that can be regionally adapted, but did not provide information about the information Internet users are most likely to be exposed to. Therefore, the objective of this study was to survey the nature of information about cognitive health that is currently available to the North American public using typical Internet search strategies and results.

## Methods

### Identification of Webpages

**Fig. 1:**
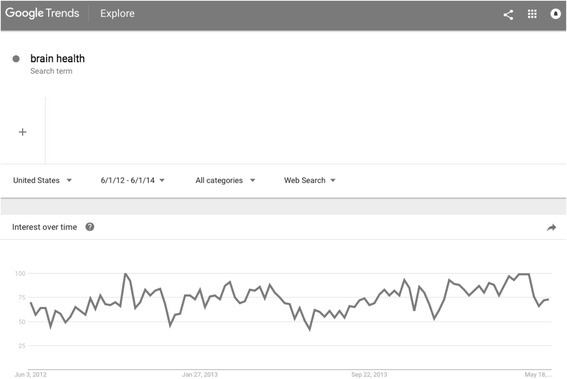
Interest in the term “brain health” in Google searches from 2012 to 2014

**Fig. 2:**
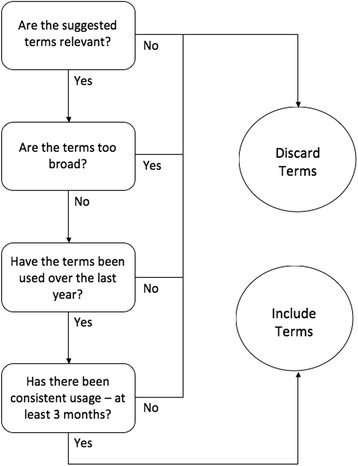
Process for adopting search terms suggested by Google Trends

Authors P.H. and J.S. initiated this review of Internet webpages by generating a list of keywords that people might use to search for information about maintaining and enhancing cognition, or about cognitive decline and dementia. These search terms were derived from a previous website review [22] (e.g., brain health, brain fitness, Alzheimer disease, memory loss) and from suggestions by the interdisciplinary research team (e.g., exercise and brain, nutrition and brain). These terms served as seeds to generate a more comprehensive list of search terms currently used by laypeople in North American Internet searches. The full list of search terms was generated by entering the seed terms in Google Trends and recording *related searches* (i.e., additional search terms). All germane results were retained, provided the terms had been used consistently in North America from June 2012 to June 2014 (see Fig. 1) and had registered above-zero search volume in May and June 2014. Any terms that did not produce germane search hits were discarded (see Fig. 2). This approach yielded a total of 155 user-generated search terms (see Additional file 1: Supplement 1).

We then continued to model our webpage search after typical Internet search behaviour. For instance, since the majority of Internet users use Google as a search engine [23–26], we selected Google as the search engine for this study. Google search results are ranked based on a number of factors, including the number of times a page has been accessed, geographical location, [27] the number of other websites linking to a particular website, [28, 29] and other indicators of website relevance and popularity [27]. Given that our objective was to employ a naturalistic search strategy, we accepted these features of the search engine as true to life. Nevertheless, we made one exception to avoid the possibility of confounding search results. Since personal browsing histories are also factored into Google search algorithms, we cleared the web browser’s history and conducted all searches with the browser set to “private”.

Research on Internet search behavior demonstrates that the average searcher views a single page of search results [30]. Generally, the first hyperlink on the page of search results is likely to be clicked [31], and the likelihood of clicking additional results decreases to 50% for the second item, about 30% for the third, and so forth [32]. In addition, about 50% of the time, Internet users who are searching for health information visit just one to three webpages after scanning this initial page of search results [33]. Therefore, to generate a set of search results approximating those an average user is most likely to encounter and explore, we entered each of our search terms in Google and recorded the first two relevant hits from the first page of search results. When multiple webpages from the same *website* surfaced in the search, we included each distinct webpage in the search results. This Google search was completed in October 2014. Search results were initially archived as webpage hyperlinks, and were subsequently archived as PDF files at the outset of the content analysis, in January 2015.

### Content analysis of Webpages

Webpage analysis was executed in three stages. First, we documented general characteristics of the webpage and corresponding website. Next, we described any content associated with cognitive health promotion or prevention. Finally, we rated the quality of the information provided on the webpage.

In order to maintain a focus on webpages rather than websites, we did not visit any hyperlinks contained within the webpages we reviewed. However, we did review the full content of web documents that were clearly intended to be read across multiple pages, as indicated by expressions such as “next”, or “page 1 of 2”.

#### Webpage and website characteristics

To document the general characteristics of the webpages, F.F. recorded the URL (Internet address) of each webpage, the date the webpage was accessed, its type (e.g., informational, shopping, discussion forum), the primary focus (i.e., cognitive health, dementia, or other), and the extent of advertising content (e.g., promotion of a specific product or company, advertising content only; or no advertising content). F.F. also documented the apparent involvement of health experts in creating the information provided on the page, and reference to national guidelines or recommendations about lifestyle strategies such as physical activity and nutrition. Finally, the website corresponding to the webpage was also reviewed in order to document the country of origin and ownership structure (e.g., commercial, not-for-profit government). P.H. confirmed these results.

#### Type of content

Next, we set out to describe whether or not each webpage contained information about promoting cognitive health, preventing risk of cognitive decline, or preventing further decline (i.e., for those with existing impairment). In order to replicate key aspects of a previous review of health system and seniors centre websites, [22] we also documented the presence or absence of information about particular strategies for cognitive health, such as diet or nutrition, physical activity, mental activity, use of technology, social involvement, and alternative medicine. During this stage of the analysis, P.H. and M.O. independently reviewed the content of 20% of the webpages and then evaluated their agreement level. The agreement level was 98% across all categories. Inter-rater reliability was then assessed within each relevant domain using Krippendorf’s alpha [34]. Resulting values of .83 to 1.0 confirmed a high level of agreement within each content domain. For the few disagreements that arose, the two raters discussed the webpage content until consensus was achieved. P.H. then completed the remainder of the content review.

#### Quality of content

The final step in our analysis was to rate the quality of information on each webpage using a 16-item standardized rating tool designed for a mixed user group of information producers, health professionals, and patients. DISCERN [35] is a fifteen-item scale used to rate the quality of published health information. The scale has previously been used in studies examining the quality of health information provided on webpages [36–39]. Scale scores are correlated with other indicators of website quality and information quality [38, 40] and show good internal consistency [40].

Eight DISCERN items are used to rate the trustworthiness (i.e., reliability) of the information presented (e.g., relevance, clarity of sources, bias) and a further seven items are used to rate information quality. The original scale focuses on quality of information for treatment choices (e.g., how the treatment works, benefits, risks), and we modified the scale to focus on prevention strategies. For example, the item, “Does it describe what would happen if no treatment is used?” was rephrased as, “Does it describe what would happen if no prevention strategy is used?” A 16th DISCERN item asked for a global rating of publication quality based on previous responses, with responses ranging from low (implying serious shortcomings) to high (implying minimal shortcomings). All items are rated using a Likert-type scale of 1 (“No”) to 5 (“Yes”) with “partially” as the neutral option.

Four of the authors (P.H., M.D., M.O, & A.C.) formed a rating team to provide DISCERN item ratings for each webpage containing content about promoting cognitive health. To ensure that all raters were well-acquainted with DISCERN, each rater independently analyzed five webpages. The rating team then met to discuss observed discrepancies and agree on consensus ratings for these webpages. The remaining 43 webpages were divided approximately equally among the rating team such that two members of the team were assigned to review each webpage independently.

We calculated inter-rater reliability using Gwet’s AC_2_ [41, 42]. This statistic was recently proposed as an alternative to more commonly used agreement coefficients, such as Cohen’s Kappa and Krippendorf’s alpha [43, 44]. Designed for ordinal data, AC_2_ can be applied when there are more than two raters. Another helpful feature of AC_2_ is that disagreements are weighted based on their seriousness [43]. The statistic is also robust in cases of high agreement, rare occurrences, or skewed data [44]. AC_2_ was calculated for every pair of raters on each of the 16 DISCERN items. A quadratic weighting was chosen for the analysis of disagreements. AC_2_ was greater than 0.7 for 12 of the 16 DISCERN items. For the four items that did not meet this threshold, AC_2_ ranged from 0.58–0.64. For each of these four items, the weighted percent agreement was 88% or higher. This indicates that we achieved acceptable levels of inter-rater reliability for most items, with marginally acceptable reliability on five items.

Nevertheless, consistent with a best practice approach to rater agreement, [45] the rating team made an a priori decision to review items with more serious disagreement closely and use a consensus-finding approach in these cases. Therefore, once all pairs of raters had completed their work, results were reviewed item-by-item. For each pair of raters, when one member’s item rating was within a single point of the other’s, the team chose to take the average of the two scores as the consensus rating. When the item rating differed more than this, the full team reviewed the webpage together and decided on a consensus rating.

We then proceeded to calculate DISCERN’s reliability and quality subscale scores. A previously reported characteristic of DISCERN is that certain questions do not apply well to some publications [35]. This issue affected just 1 in 1290 items in our dataset. In this instance, we calculated DISCERN subscale scores dividing the sum of ratings by the number of items completed to arrive at a prorated score [46].

Although content expertise is not required for DISCERN ratings, [35] raters were asked to respond to an additional item that did rely on content expertise (P.H., M.D., M.O, & A.C. are trained health and social scientists with respective expertise in clinical psychology; geriatric clinical neuropsychology; health product marketing; and clinical dietetics and nutrition science). The additional item, once again rated on a 1 to 5 (poor to excellent) Likert-type scale, was: *What is your global rating of the quality of evidence on this page?* Gwet’s AC_2_ was .70, suggesting acceptable agreement overall. Once again, the team reviewed discrepant scores to improve agreement. When discrepancies were within one point, the average of the two raters’ scores as the final score; otherwise, a consensus-finding approach was used.

## Results

**Fig. 3:**
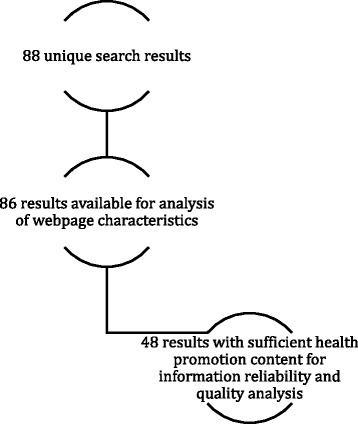
Search results

The Google search generated 88 webpages; however, two of these became unavailable prior to archiving, reducing the total obtained sample from 88 to 86 (see Fig. 3 and Additional file 1: Supplement 2).

### Web page and site characteristics

For the obtained sample of 86 webpages, webpage and corresponding website characteristics, including country of origin, ownership, webpage type, primary focus, type of advertising content, expert involvement, and reference to consensus guidelines, are summarized in Table 1. As Table 1 further illustrates, the majority of pages (83.7%) that surfaced in the search were from the United States of America, and the remainder were from Canada, the United Kingdom, and Australia. Although several pages (22.1%) made reference to experts or indicated direct expert involvement in the construction of the article, basic information that would help to position the individual as an expert (i.e., name, credentials, and institutional affiliation) was given for only 3.5% of the pages we reviewed.

**Table 1:** Characteristics of the websites associated with a sample of 86 webpages

Characteristics	Number (%) of websites
Country of origin:	
United States of America	72 (83.7)
Canada	8 (9.3)
United Kingdom	5 (5.8)
Australia	1 (1.2)
Website ownership structure:	
Commercial	39 (45.3)
Not-for-profit	38 (44.2)
Government	5 (5.8)
University	4 (4.7)
Personal	0 (0)
Webpage type:	
Information	66 (76.7)
Wiki	8 (9.3)
Scholarly work	4 (4.7)
Health product	5 (5.8)
News	2 (2.3)
Discussion forum	1 (1.2)
Primary focus of webpage:	
Cognitive health	41 (47.7)
Dementia	34 (39.5)
Other	11 (12.8)
Advertising content on webpage:	
Promotion of a specific product or company	10 (11.6)
Mixed advertising content on page	29 (33.7)
No advertising content	47 (54.7)
Webpage indicates expert involvement:	
Name, credentials, and affiliation noted	3 (3.5)
Name and credentials noted	16 (18.6)
Name noted	16 (18.6)
No reference to experts	67 (77.9)
Webpage refers to consensus guidelines:	
Yes	1 (1.2)
Unnamed	2 (2.3)
No	84 (97.7)

Note: Two of the original pages were no longer available when these characteristics were summarized in July 2015, resulting in a sample of 86 webpages

Of the 86 webpages analyzed, 4 (4.7%) were from universities, 5 (5.8%) were from government organizations, 38 (44.2%) were from not-for-profit organizations, and the remaining 39 (45.3%) were from commercial organizations. The majority of the commercial webpages (33.7%) were *content providers*; that is, webpages that provide information on health-related topics alongside paid advertising and sponsored content. The website webMD.com is a prime example of such a webpage. The remaining commercial webpages (11.6%) were *product promoters*; that is, webpages that promote products or services for a particular brand or company. For instance, included among these were webpages promoting Dr. Perlmutter’s books on the alleged benefits of grain-free diets.

Pages were most frequently formatted as information pages, and less frequently as wikis, scholarly works, news articles, or discussion forums. For additional details, see Table 1.

### Type of content

Of the 86 surveyed pages, 41 (47.7%) had a general focus on brain health, 34 (39.5%) on dementia, and 11 (12.8%) had another focus (e.g., ADHD, cognition, nutrition, etc.; see Table 1). The majority of retrieved webpages (65.1%) contained information about either promoting cognitive health or preventing cognitive decline (see Table 2). Only 16.3% of sites provided information about the possibility of preventing further cognitive decline.

**Table 2:** Level of information about preventing cognitive decline or promoting cognitive health within a sample of 86 webpages

Web page characteristic	Yes (%)	No (%)
Any information on promoting cognitive health	38 (44.2)	48 (55.8)
Any information about preventing cognitive decline	37 (43.0)	49 (57.0)
Any information on either prevention or promotion	56 (65.1)	30 (34.9)
Any information about preventing *further* decline	14 (16.3)	72 (83.7)
Specific information about promoting cognitive health *and/or* preventing decline	48 (55.8)	38 (44.2)

Thirty-five webpages (40.7%) offered multiple strategies to prevent cognitive decline or promote cognitive health. Of these, diet and nutrition were most frequently mentioned (48.8%), followed by physical activity (30.2%), and then mental activity (29.1%). Vitamins, computerized “brain games,” and social involvement were each identified as potential strategies on 15% or fewer of the sites (see Table 3). Other commonly referenced strategies on reviewed sites included smoking cessation, reduction of alcohol consumption, head protection, stress reduction (including meditation and yoga), adequate sleep, management of diabetes, management of depression, and management of cardiovascular risks.

**Table 3:** Identification of specific strategies to prevent cognitive decline or promote cognitive health in a sample of 86 webpages

Strategy	Yes (%)
Diet and nutrition	42 (48.8)
Physical activity	26 (30.2)
Mental activity	25 (29.1)
Alternative medicines, herbs, medical foods, vitamins	13 (15.1)
Brain games or specific electronic interfaces	12 (14.0)
Social involvement	11 (12.8)
Other (e.g., smoking cessation, head protection)	18 (20.9)

Just one site (1.2%) mentioned best practice and/or consensus guidelines by name. In this case, the American Heart Association’s exercise guidelines were mentioned. Two additional sites (2.3%) made generic reference to dietary allowances or recommended servings of fruits and vegetables (see Table 1).

### Quality of content

We limited the number of webpages subjected to quality analysis to those identified as containing specific information about promoting cognitive health or delaying cognitive decline (see Table 2). This resulted in a total sample of 48 websites (38 were excluded at this stage; see Fig. 3). Subject matter expert (SME) global ratings of the quality of evidence across the 48 webpages averaged 2.39 (*SD* = 1.20). The average DISCERN information reliability subscale score across the 48 webpages was 3.10 (*SD* = .87), with 3 being the midpoint rating on the DISCERN scale. The average DISCERN information quality subscale score across these pages was 2.40 (*SD* = .73), which was below the midpoint on the scale. The global rating of the pages as a source of information about prevention or promotion strategies related to cognitive health, using DISCERN’s 16th (standalone) item, was 2.63 (*SD* = 1.05). This information is also summarized in Table 4.

**Table 4:** Average DISCERN score for 48 webpages

Scale	Subscale or item	Mean (SD)
DISCERN	Information reliability subscale	3.10 (0.87)
	Information quality subscale	2.40 (0.73)
	Global rating of the overall quality of the publication as a source of information about prevention strategies	2.63 (1.05)
Other	Global rating of the quality of evidence provided in the publication	2.39 (1.20)

Note: From the full sample of 86 webpages, only webpages containing substantive information about cognitive health promotion or prevention of cognitive decline are included here (*N* = 48)

Table 5 provides a list of the top-ranking webpages by information reliability, information quality, and SME ratings, respectively. Despite accounting for just 35.4% of this subsample of 48 webpages, non-commercial webpages accounted for half or more of the top-rated webpages in terms of reliability, quality and SME ratings (63.6%, 60.0% and 75.0% respectively).

**Table 5:** Top 10 webpages ranked by information reliability, information quality, and subject matter expert ratings

Rank	By information reliability	Score	By information quality	Score	By subject matter expert rating	Score
1	https://www.alz.co.uk/sites/default/files/pdfs/nutrition-and-dementia.pdf	4.94	https://www.alz.co.uk/sites/default/files/nutrition-and-dementia-introduction.pdf	3.93	https://www.alz.co.uk/sites/default/files/pdfs/nutrition-and-dementia.pdf	5.00
2	http://www.ncbi.nlm.nih.gov/pmc/articles/PMC2805706/	4.69	https://www.alz.co.uk/sites/default/files/pdfs/nutrition-and-dementia.pdf	3.92	http://en.wikipedia.org/wiki/Alzheimer's_disease	5.00
3	http://en.wikipedia.org/wiki/Alzheimer‘s_disease	4.69	http://www.webmd.com/vitamins-and-supplements/lifestyle-guide-11/fortifying-your-memory-with-supplements	3.71	https://www.alz.co.uk/sites/default/files/nutrition-and-dementia-introduction.pdf	4.50
4	https://www.alz.co.uk/sites/default/files/nutrition-and-dementia-introduction.pdf	4.63	http://www.ncbi.nlm.nih.gov/pmc/articles/PMC2805706/	3.64	http://www.ncbi.nlm.nih.gov/pmc/articles/PMC2805706/	4.50
5	http://www.medicalnewstoday.com/articles/142214.php	4.56	http://www.healthline.com/health/dementia/vitamins-memory-loss#Overview1	3.5	http://www.alz.org/we_can_help_brain_health_maintain_your_brain.asp	4.00
6	http://en.wikipedia.org/wiki/Brain_fitness	4.31	http://hbr.org/2007/11/cognitive-fitness/ar/1	3.18	http://www.alz.org/we_can_help_adopt_a_healthy_diet.asp	4.00
7	http://www.brainhealth.utdallas.edu/blog_page/study-finds-aerobic-exercise-improves-memory-brain-function-and-physical-fi	4.19	http://www.healthybrainforlife.com/articles/school-health-and-nutrition/feeding-the-brain-for-academic-success-how [now defunct]	3.07	http://www.brainhealth.utdallas.edu/blog_page/study-finds-aerobic-exercise-improves-memory-brain-function-and-physical-fi	4.00
8	https://en.wikipedia.org/wiki/Neurobiological_effects_of_physical_exercise	4.06	http://www.helpguide.org/articles/alzheimers-dementia/alzheimers-and-dementia-prevention.htm	3.00	http://en.wikipedia.org/wiki/Brain_fitness	4.00
9	http://www.healthline.com/health/dementia/vitamins-memory-loss#Overview1	3.94	http://www.helpguide.org/articles/memory/how-to-improve-your-memory.htm	3.00	http://www.medicalnewstoday.com/articles/142214.php	4.00
10	http://www.alz.org/we_can_help_adopt_a_healthy_diet.asp	3.88	http://www.memory-improvement-tips.com/vitamins-for-memory.html	3.00	http://www.alz.org/what-is-dementia.asp	3.50
11	http://www.health.com/health/gallery/0,,20434658,00.html	3.88			http://www.healthline.com/health/dementia/vitamins-memory-loss#Overview1	3.50
12					http://www.health.com/health/gallery/0,,20434658,00.html	3.50

Note: Several webpages are equally ranked, resulting in lists of 10–12 webpages, listed here in alphabetical order. From the full sample of 86 webpages, only webpages containing substantive information about cognitive health promotion or prevention of cognitive decline were considered in these rankings (*N* = 48)

#### Relationship between quality of content and webpage characteristics

Based on our subjective impressions of webpage content, we hypothesized statistically significant differences in the quality, reliability, and SME ratings across content provider, product promoter, and non-commercial webpages, and that these differences would favour content provider and non-commercial webpages over product promoter webpages. We used a series of one-way ANOVA to test this hypothesis.

In the first ANOVA, reliability of information was the dependent variable, and owner type (content provider, product promoter, or non-commercial) was the independent variable (factor). Reliability differed by owner type (*F* = 12.01, *p* < 0.05), and post-hoc comparisons revealed that non-commercial webpages rated significantly more reliable than content provider webpages (*M*_non-comm_ = 3.6 vs. *M*_content_ = 3.0, *p* < .05) as well as product promoter webpages (*M*_non-comm_ = 3.6 vs. *M*_product_ = 1.9, *p* < .05). There was also a significant difference in reliability between content providers and product promoters; content providers were rated as significantly more reliable than product promoters (*M*_content_ = 3.0 vs. *M*_product_ = 1.9, *p* < .05).

Similarly, a one-way ANOVA with subject-matter expert rating of evidence quality as the dependent variable and owner type as the factor showed that evidence quality differed by owner type (*F* = 10.8, *p* < .05). Non-commercial webpages were rated significantly higher than both content provider (*M*_non-comm_ = 3.2 vs. *M*_content_ = 2.1, *p* < .05) and product promoter webpages (*M*_non-comm_ = 3.2 vs. *M*_product_ = 1.2, *p* < .05). Content provider webpages, however, were not rated significantly higher than product promoter webpages (*M*_content_ = 2.1 vs. *M*_product_ = 1.2, *p* > .05).

A different pattern emerged when comparing the three types of webpages in terms of quality ratings. A one-way ANOVA with information quality as the dependent variable and owner type as the factor showed that information quality differed by owner type (F = 5.28, *p* < .05), but post-hoc comparisons revealed that the only significant difference in quality ratings was between non-commercial and product promoter webpages (*M*_non-comm_ = 2.79 vs. *M*_product_ = 1.8, *p* < .05). There were no significant differences between the non-commercial and content-provider webpages (*M*_non-comm_ = 2.79 vs. *M*_content_ = 2.27, *p* = .05) or between the content-provider and product promoter webpages (*M*_content_ = 2.2 vs. *M*_product_ = 1.8, *p* > .05).

## Discussion

This review of webpages, compiled using a naturalistic search strategy for information about strategies to promote brain health, has demonstrated that, for the most part, Internet publishers did not “get it right”. Based on scores on a standardized measure, the vast majority of cognitive health promotion webpages considered in this review did not present reliable, high quality information to readers. These findings are of critical importance since they represent the information available to most individuals who are seeking to teach themselves about cognitive health.

### The range of cognitive health promotion strategies observed

A prior review of cognitive health information on the Internet focused on the content of health care system, public health department, and senior centre websites [22]. This review found that health care systems were more likely to provide cognitive health promotion and prevention content than public health departments and seniors centres. Nutritious eating, physical activity, and mental activity were each promoted on approximately one fifth of health care system websites, and social activity was promoted on approximately 16% of websites. In the current review, the pattern of results differed, with nutritional strategies surfacing in nearly 50% of websites, mental and physical activity in about 30%, and social activity in 11%. ‘Brain games’ and dietary supplements also surfaced regularly in our review, though not in the prior one. The divergent content of health system and general Internet websites raises some questions about the preponderance of information about ‘brain games’, dietary supplements, and nutrition-related information in the general Internet search. It is plausible that health system websites provide a more cautious and balanced application of the evidence base than websites retrieved using a general Internet search.

Physical activity and exercise is the most robustly supported strategy to mitigate the impacts of cognitive aging, [47] reduce risk of mild cognitive impairment, and delay onset of dementia, [48, 49] particularly after mild cognitive impairment [50, 51]. In fact, it is the primary non-pharmacologic intervention for persons with dementia [52, 53]. Although many webpages we reviewed presented information on multiple strategies to support cognition, physical activity was only referenced in approximately 30% of these.

Still, we were encouraged to see that nearly half of the webpages we reviewed presented multiple strategies for promoting cognitive health and delaying decline. This trend is consistent with emerging evidence that a number of lifestyle strategies may help to slow cognitive decline [1, 2] and that genetic predispositions may confer differential advantages for some of these over others, depending on the individual [3, 4]. Given the present state of the evidence, we expect that the next several years will see Internet publishers with interests in cognitive health redouble their efforts to present multiple options for promoting cognitive health and delaying decline.

### Information reliability and quality

Exercise and nutrition were the most frequently mentioned strategies for maintaining cognitive health or delaying decline. Surprisingly, although consensus and/or best practice recommendations for exercise and nutrition are in place in each of the countries from which our pool of webpages originated, [54–58] only one webpage specifically referred to such guidelines. Among the eight DISCERN items we used to assess the reliability of information presented on the webpages we reviewed, one inquired about the sources used to generate the information, another assessed whether other sources of information are offered, and a third evaluated the level of bias in the information presented. Citing available consensus guidelines would be a very useful way for health information publishers to address each of these indicators of information reliability.

Recommendations concerning diet and food surfaced in nearly half of the sampled websites, far more often than any other health promotion strategy. Although the Mediterranean diet and other high-nutrition diets show potential as a cognitive health strategy, [59–66] we found that the vast majority of the nutrition-related webpages that surfaced in our search contained inaccurate recommendations or other misinformation. This is worrisome, given that 51% of people use the Internet as a source of nutrition information, and many perceive Internet sources to be credible [67]. Of all webpages generated in our search, pages containing a focus on diet and nutrition were most likely to contain advertising content, even when the pages were not explicitly designed to market products.

### Product promoter sites and marketing ethics

Webpages designed to sell a product or promote a company (i.e., product promoters) represented approximately 11.6% of our webpage sample. Most of these product promoter sites that surfaced in our search marketed nutrition products and “brain games”. This very specific focus is in contrast with a more complex message derived from research on the efficacy and effectiveness of such products. For example, vitamin-rich diets are recommended over single vitamin supplements or nutrition products for mitigating effects of cognitive aging [58, 68]. Furthermore, although several studies show improvement in the cognitive skills directly exercised by ‘brain games’ or computerized cognitive training, [69] there is significant controversy as to whether these skills are generalizable to everyday life [70]. or lasting [71]. Put succinctly, “while data is promising and negative side effects from computer-based cognitive enhancement strategies are likely minimal, the current base of scientific evidence is insufficient to support many commercial claims of the efficacy of their computerized cognitive enhancement systems” [72].

As more and more people turn to the Internet for health-related information, the number of commercial webpages is not likely to diminish. One troubling aspect of our results has to do with webpages promoting a specific product or company. These pages scored consistently lower than non-commercial webpages in terms of both reliability and quality. Whereas content providers fund their operations by selling advertising space and sponsored content but provide diverse information, product promoter webpages are entirely devoted to selling one particular solution and viewpoint of a health condition. Regulators might want to be especially vigilant in monitoring product promoters’ claims. For example, the ‘brain training’ program Lumosity was marketed with claims of benefits such as improved performance in school, work, and sport, and protection against cognitive decline, all said to be evidence-based [73]. The US Federal Trade Commission (FTC) raised charges against the parent company, Lumos Labs, alleging that these claims deceived consumers. In January 2016, as a result of the complaint, Lumos Labs received a $50 million judgment and was reminded to rely on rigorous scientific evidence to make claims about any everyday or health benefits associated with their product. Internet-based claims about Lumosity now appear to be much more objective. For instance, their homepage presently states, “we need to do more research to determine the connection between improved assessment scores and everyday tasks in participants’ lives” [74].

Marketing ethicists encourage marketers to go beyond the law in their ethical responsiveness, and to ensure their activities act to benefit rather than harm consumers and society at large [75, 76]. A core set of ethical principles for marketing has been proposed, and includes non-malfeasance, non-deception, protection of vulnerable market segments, distributive justice, and social stewardship [75]. These principles are directly relevant to Internet-based marketing of information or products designed to promote cognitive health:

1. The first principle, non-malfeasance, encourages marketers to avoid knowingly doing harm. Although many of the products that surfaced in our search may not have important benefits, most are unlikely to cause harm. Nevertheless, some products that surfaced in our search, including restricted diets and dietary supplements, do have risks attached [66].
2. According to the second principle, non-deception, marketers should emphasize trust and avoid both blatant deception and misleading consumers. For instance, Lumos Labs was found to have misled consumers to increase adoption of Lumosity. Careful attention to this ethical principle could have promoted consumer interests and prevented the legal action against the company.
3. The third principle emphasizes protecting vulnerable segments of the market against exploitation. Generally, consumers have difficulty assessing the quality of claims associated with neuroscience [77]. Having a health or mental health condition may contribute to additional vulnerability [77]. For instance, older adults who have compromised mental and cognitive health also tend to have lower health literacy, which may increase risk of exploitation in health-oriented marketing [78].
4. An additional ethical principle is distributive justice, which emphasizes fair distribution of goods. Although Internet may not be regularly accessible to all North American consumers, it is accessible to the grand majority. Internet-based marketing thus enhances fairness (albeit imperfectly) by providing access to information and products designed to promote cognitive health across geographic regions and a number of social strata.
5. A final ethical principle for marketers is that of stewardship, which recognizes that profit motivations should not result in long-term costs to society. The principle of stewardship can be extended to include the potential social costs of propagating misinformation. For instance, it is likely that professional time is being expended to correct misinformation at public expense.

These principles overlap and intersect. For instance, as a result of the misleading use of science to promote Lumosity, some consumers may not have searched for other, more beneficial resources. Since Lumos Labs marketed their product to vulnerable clients (e.g., people who were at greater risk for cognitive impairment; people with specific health conditions), [73] the misleading information may have been more consequential, perhaps even violating the principle of non-malfeasance. As clinicians’ efforts to provide balanced information to potentially vulnerable patients were thwarted by overstated claims that Lumosity was backed by science, the company wasted and undermined social resources, violating the principle of social stewardship.

### Are all commercial sites suspect?

Our analysis showed that product promoter webpages, one type of commercial webpage, scored lower on DISCERN’s reliability and quality subscales than content provider webpages and non-commercial webpages. Nevertheless, content provider webpages, another type of commercial webpage, did not differ from non-commercial webpages on DISCERN’s information quality subscale. Considering the high percentage of commercial webpages returned in our search, this is an important finding. While the reliability of the content provider webpages could be improved (e.g., by crediting sources), at least the quality of the information people are being exposed to while searching for health-related topics on commercial content provider webpages (e.g., WebMD.com) seems to be on par with that provided by not-for-profit organizations.

### Could commercial sites be superior in any way?

A recent study examined user perceptions of website quality when searching for information on a particular health topic [79]. The study compared hospital websites and commercial content provider websites such as WebMD, and found that users had significantly more favourable attitudes toward the commercial websites. This suggests that some characteristics of content provider websites are compelling to users.

Although we did not formally assess the readability and usability of the webpages that surfaced in our study, we noted a tendency for more comprehensive articles (e.g., Wikipedia and academic articles; see Table 5) to receive higher reliability scores, and wondered whether Internet users would expend the time and effort required to mine these lengthy articles for useful information. In keeping with this, health communications researchers have observed that higher quality content correlates with lower readability and reduced usability [80]. Besides improving access to high-quality, reliable information about brain health, website readability and usability also deserve consistent attention if this information is to attract the attention of the average Internet user [81–85]. Moreover, it may also be important to assess whether people searching for information about cognitive health are indeed average users, and to design websites to suit the intended audience. For instance, one recent study demonstrated that older adults find websites organized around keyword tags easier to navigate than hierarchically organized websites [86]. Since cognitive capacity is also known to interact with website usability, [87] website designers should also consider the needs of special populations [88].

### Research Strengths & Limitations

This study has some important strengths and limitations. Methodological strengths of the study include employment of a naturalistic search strategy to approximate user results and behaviour, use of a standardized measure of information quality, use of a consensus rating process to generate webpage ratings, and resolution of rater discrepancies through a thorough review and discussion.

Our methods to enhance generalizability were imperfect. For instance, given that the Google search algorithm includes geography as one of hundreds of variables that influences search results, our locale may reduce the replicability of our sample of webpages. Furthermore, Internet websites are constantly being updated for currency, and webpage presence and content may change quickly over time. Similarly, search results are likely to change based on social trends. Thus, the study is best conceptualized as a snapshot in time from a particular vantage point.

Finally, to ensure a focus on cognitive health, we evaluated webpages rather than websites. Thus, we did not evaluate the overall quality of health information available on websites such as Wikipedia, WebMD.com, Mayo Clinic, or Alzheimer Society. We did, however, evaluate the quality of information about cognitive health provided on webpages associated with these sites. Since a review of the full websites is likely to lead to somewhat different conclusions about information quality or reliability, it is important that our results not be over-generalized.

### Future directions

This study relied on a unimodal search engine based strategy to represents health information searches on the Internet. However, it is also possible some health-conscious searchers access information from the Internet without using search engines. For instance, people may search for Smartphone applications instead of or in addition to reviewing websites, [89, 90] and these searches typically take place within an application store. In addition, researchers have begun to explore health information dissemination on social media platforms such as Facebook and Twitter [91]. Although the most recent available research on this suggests that search engines do remain the starting point for health information searches, [32] given the pace of change on the Internet, this is something that should receive sustained research attention.

In ongoing research on health information searching, the interaction of time and search behaviour should also be further explored. For instance, it is possible that some health-conscious searchers come to favour sites that surface frequently in their searches, such as those listed in Table 5, and eventually begin their searches at these sites, rather than in a search engine, or refine their searches to include the name of the health website. Further, some recent research suggests that health information searches are systematic, with consistencies in general themes but variance in specific keywords or strategies seen over the span of several days [92]. There is also some evidence that health information searching is multi-modal and includes in vivo interactions with health care providers, [93, 94] which subsequently help to determine the content of Internet searches [92].

### Recommendations for the busy clinician

Since searching for health information on the Internet is the rule rather than the exception, there is no point in wishing it away. In contrast, busy clinicians should work to cultivate an approach that includes:

- **Empathy**. Work to understand why patients are mentioning Internet sources, and respond in a manner that aligns with their questions and motivations.
- **Education**. Patients may not realize that the polarized and distilled way that scientific results are communicated on the Internet differs greatly from the balanced and qualified approach adopted in reputable scientific journals [95]. They can be taught to look for positive indicators of trustworthiness, such as reference to scientific evidence or national guidelines. They can also be taught to be more suspicious of claims made on webpages used to market products, or of scientific claims made by people with financial interest in a company [77]. Clinicians can also identify websites they are comfortable recommending (e.g., see Table 5).
- **Hope**. Many patients believe unsubstantiated claims because they give hope. Rather than leaning toward the extremes of confrontation or avoidance, we urge clinicians to state the facts, and foster hope by offering evidence-based alternatives such as physical activity and other healthy lifestyle choices. Clinicians can also remind a patient that since each of us has a distinct genetic heritage, if there is any “one size fits all” strategy to improve cognitive health, it is to achieve balance across a number of simple, healthy lifestyle choices, including regular exercise, a nutritious diet, and personally engaging activities [5–8]. Again, some currently available sites do this well (see Table 5).
- **Respect for autonomy**. Even when a claim is not substantiated by research, there may be some personal value in pursuing it, such as having fun, being trendy, or expressing autonomy. Clinicians need not dissuade patients from these pursuits so long as there is no likelihood of harm. However, it may be useful to revisit these ideas over time, introducing evidence-based alternatives as enthusiasm wanes.

## Conclusion

These results are offered as a baseline assessment of cognitive health promotion efforts on the Internet, in anticipation of national and global efforts to increase availability of information about the effects of lifestyle on cognitive health. This review of webpages, compiled using a naturalistic search strategy for information about strategies to promote brain health, has demonstrated that the vast majority of Internet publishers did not “get it right”. As a compelling example of this, only one webpage that surfaced in our search made direct reference to consensus guidelines, despite that a number of relevant guidelines are available. Additional work must be done to promote high-quality, reliable information about cognitive health on the Internet. For busy clinicians negotiating these issues with their patients, we recommend warning patients that webpages designed to promote products are more likely than other webpages to provide misleading information, and showing patients how to search for evidence-based guidelines.

## Additional file

Additional file 1: Supplements 1 & 2. (DOCX 36 kb)
