# Supplementary material for: Did online publishers “get it right”? Using a naturalistic search strategy to review cognitive health promotion content on internet webpages
Source: BMC Geriatr. 2017 Jun 15;17:125. doi: 10.1186/s12877-017-0515-3 (PMC5472889; doi:10.1186/s12877-017-0515-3)
Supplement: Additional file 1: — Supplements 1 & 2. (DOCX 36 kb) [file 12877_2017_515_MOESM1_ESM.docx]

**Supplement 1**

To arrive at the following list of search terms, seed search terms related to cognitive health (e.g., “cognitive health”, “brain health”) were entered in Google Trends during July and August 2014. All germane results generated by Google Trends were added to this list, provided these terms had been used consistently in North America for at least two years, and had registered above-zero search volume over the most recent two months.

activities and dementia

activities dementia patients

activities for dementia

aerobic exercise benefits

Alzheimer disease

alzheimer's

alzheimers dementia

alzheimers disease

alzheimers symptoms

benefits of diets

benefits of exercise

benefits of fitness

benefits of nutrition

best brain food

best brain foods

body mind nutrition

brain and diet

brain diet

brain exercise

brain exercise games

brain exercises

brain fitness

brain fitness program

brain food

brain food diet

brain food memory

brain food recipes

brain foods

brain grain diet

brain gym

brain gym activities

brain gym exercise

brain health

brain healthy diet

brain healthy food

brain healthy foods

brain power foods

brain vitamin

care for dementia

care for dementia

care for elderly

care of elderly

care plan dementia

causes of dementia

cognitive fitness

cognitive health

cognitive mental health

components of fitness

components of health

components physical fitness

definition of fitness

dementia

dementia activities

dementia and treatment

dementia care

dementia diet

dementia signs

dementia symptoms

dementia symptoms early

dementia treatment

diet and brain

diet and dementia

diet for brain

diet for dementia

early dementia

early dementia signs

elderly and healthcare

elderly care

elderly health care

elderly healthcare

emotional fitness

exercise and brain

exercise and memory

exercise your brain

feed your brain

fitness and brain

fitness components

food and brain

food and memory

food for brain

food for memory

food improve memory

foods for brain

foods for memory

good brain food

good brain foods

good memory food

grain brain

grain brain diet

health components

health diet benefits

health related components

health related fitness

healthcare for elderly

healthy brain food

healthy brain foods

improve memory foods

loss of memory

memory exercises

memory food

memory foods

memory loss

memory loss causes

memory loss disease

memory loss foods

memory loss problems

memory loss symptoms

memory loss treatment

memory problems

mental fitness

mental health fitness

mental health nutrition

mind exercise

mind exercises

mind foods

mind nutrition

nutrition and brain

nutrition and dementia

nutrition benefits

physical fitness benefits

physical fitness definition

posit science

posit science brain

preventative care

preventative health

preventative health care

preventative health services

preventive care

preventive health

preventive health care

preventive health services

preventive services

problems with memory

short memory loss

short term memory loss

signs of alzheimers

signs of dementia

social fitness

successful aging

supplements for memory

symptoms of alzheimers

symptoms of dementia

The brain diet

treatment for dementia

treatment of dementia

vitamin for brain

vitamin for memory

vitamins for brain

vitamins for concentration

vitamins for memory

what causes dementia

what is alzheimer

what is alzheimers

what is dementia

what is fitness

**Supplement 2**

http://hbr.org/2007/11/cognitive-fitness/ar/1

http://en.wikipedia.org/wiki/Brain_fitness

http://www.brainhq.com/brain-resources/everyday-brain-fitness

http://www.brainhq.com/brain-resources/everyday-brain-fitness/physical-exercise

http://www.brainmetrix.com/

http://www.huffingtonpost.com/dennis-kravetz/10-ways-to-boost-your-cognitive-fitness_b_3195153.html

http://www.webmd.com/fitness-exercise/features/train-your-brain-with-exercise

http://www.wikihow.com/Exercise-Your-Brain

http://en.wikipedia.org/wiki/Alzheimer's_disease

http://www.alz.org/we_can_help_brain_health_maintain_your_brain.asp

http://www.alz.org/what-is-dementia.asp

http://www.alzheimer.ca/en/About-dementia/About-the-brain/Brain-health

http://www.helpguide.org/life/prevent_memory_loss.htm

http://www.livescience.com/42891-short-term-memory-loss.html

http://www.mayoclinic.org/diseases-conditions/dementia/basics/causes/con-20034399

http://www.medicalnewstoday.com/articles/142214.php

http://www.webmd.com/brain/memory-loss

https://en.wikipedia.org/wiki/Neurobiological_effects_of_physical_exercise

http://www.brainhealth.utdallas.edu/blog_page/study-finds-aerobic-exercise-improves-memory-brain-function-and-physical-fi

http://healthyeating.sfgate.com/5-benefits-proper-nutrition-3664.html

http://www.alz.org/we_can_help_adopt_a_healthy_diet.asp

http://www.bbcgoodfood.com/howto/guide/10-foods-boost-your-brainpower

http://www.besthealthmag.ca/eat-well/nutrition/foods-that-fight-memory-loss

http://www.drperlmutter.com/5-keys-eating-better-brain-healthp/?hvid=2axZqP

http://www.drperlmutter.com/about/grain-brain-by-david-perlmutter/

http://www.drperlmutter.com/learn/faq/what-is-the-ideal-diet-for-optimal-brain-function/

http://www.eatingwell.com/nutrition_health/healthy_aging/eat_for_a_sharper_mind_5_brain_boosting_foods

http://www.eatingwell.com/nutrition_health/healthy_aging/foods_that_boost_brain_power

http://www.foodnetwork.com/healthy/photos/6-foods-to-fuel-your-brain.html

http://www.health.com/health/gallery/0,,20434658,00.html

http://www.healthline.com/health/dementia/vitamins-memory-loss#Overview1

http://www.healthybrainforlife.com/articles/school-health-and-nutrition/feeding-the-brain-for-academic-success-how

http://www.helpguide.org/articles/alzheimers-dementia/alzheimers-and-dementia-prevention.htm

http://www.helpguide.org/articles/memory/how-to-improve-your-memory.htm

http://www.livestrong.com/article/87671-vitamins-focus-concentration/

http://www.medicinenet.com/script/main/art.asp?articlekey=55784

http://www.memory-improvement-tips.com/vitamins-for-memory.html

http://www.ncbi.nlm.nih.gov/pmc/articles/PMC2805706/

http://www.prevention.com/food/healthy-eating-tips/best-foods-your-brain

http://www.rodalenews.com/brain-foods

http://www.webmd.com/add-adhd/guide/vitamins-supplements-adhd

http://www.webmd.com/diet/features/eat-smart-healthier-brain

http://www.webmd.com/vitamins-and-supplements/lifestyle-guide-11/fortifying-your-memory-with-supplements

https://faculty.washington.edu/chudler/nutr.html

http://cas.umkc.edu/casww/sa/whatissa.htm

https://en.wikipedia.org/wiki/Successful_aging

http://www.cdc.gov/aging/healthybrain/

https://en.wikipedia.org/wiki/Brain_Fitness_Program

https://en.wikipedia.org/wiki/Cognitive_behavioral_therapy

https://en.wikipedia.org/wiki/Cognitive_disorder

http://heroicimagination.org/research/social-fitness/

http://www.brainhq.com/

http://www.brainhq.com/brain-training-products/brain-fitness-program

http://www.brightfocus.org/alzheimers/resources/memorygames.html

http://www.cmha.ca/mental-health/your-mental-health/

http://www.gamesforthebrain.com/

http://www.lumosity.com/

https://www.nia.nih.gov/health/featured/memory-cognitive-health

http://alzheimers.org.uk/Caring_for_someone_with_dementia/

https://en.wikipedia.org/wiki/Dementia

http://www.alz.org/alzheimers_disease_10_signs_of_alzheimers.asp

http://www.alz.org/alzheimers_disease_know_the_10_signs.asp

http://www.alz.org/alzheimers_disease_stages_of_alzheimers.asp

http://www.alz.org/alzheimers_disease_what_is_alzheimers.asp

http://www.alz.org/care/alzheimers-dementia-activities.asp

http://www.alz.org/care/dementia-creating-a-plan.asp

http://www.alz.org/dementia/types-of-dementia.asp

http://www.alz.org/living_with_alzheimers_101_activities.asp

http://www.alzheimer.ca/en/About-dementia/Alzheimer-s-disease/Warning-signs-and-symptoms/10-warning-signs

http://www.alzheimer.ca/en/About-dementia/Alzheimer-s-disease/What-is-Alzheimer-s-disease

http://www.alzheimer.ca/en/About-dementia/Dementias/What-is-dementia

http://www.caring.com/articles/activities-for-dementia-alzheimers-patients

http://www.helpguide.org/elder/alzheimers_dementias_types.htm

http://www.helpguide.org/elder/alzheimers_disease_dementias_caring_caregivers.htm

http://www.helpguide.org/life/improving_memory.htm

http://www.mayoclinic.org/diseases-conditions/alzheimers-disease/in-depth/memory-loss/art-20046326

http://www.nhs.uk/Conditions/dementia-guide/Pages/dementia-treatment.aspx

http://www.webmd.com/alzheimers/tc/dementia-treatment-overview

https://caregiver.org/caregivers-guide-understanding-dementia-behaviors

https://fightdementia.org.au/support-and-services/i-care-for-someone-with-dementia/activities-for-people-with-dementia

http://www.cdc.gov/healthyyouth/nutrition/facts.htm

http://www.dietitians.ca/Dietitians-Views/Health-Care-System/Mental-Health.aspx

http://www.doctoroz.com/article/brain-diet

http://www.drperlmutter.com/about/grain-brain-cookbook/

http://www.eatingwell.com/recipes_menus/recipe_slideshows/brain_boosting_dinner_recipes

http://www.healthline.com/health/5-benefits-healthy-habits

https://www.alz.co.uk/sites/default/files/nutrition-and-dementia-introduction.pdf

https://www.alz.co.uk/sites/default/files/pdfs/nutrition-and-dementia.pdf
